# A metagenomic-based survey of microbial (de)halogenation potential in a German forest soil

**DOI:** 10.1038/srep28958

**Published:** 2016-06-29

**Authors:** Pascal Weigold, Mohamed El-Hadidi, Alexander Ruecker, Daniel H. Huson, Thomas Scholten, Maik Jochmann, Andreas Kappler, Sebastian Behrens

**Affiliations:** 1 Geomicrobiology, Center for Applied Geosciences, University of Tuebingen, Germany; 2 Algorithms in Bioinformatics, Center for Bioinformatics, University of Tuebingen, Germany; 3 Soil Science and Geomorphology, Geography, University of Tuebingen, Germany; 4 Instrumental Analytical Chemistry, Faculty of Chemistry, University of Duisburg-Essen, Germany; 5 Department of Civil, Environmental, and Geo- Engineering, University of Minnesota, MN, USA; 6 BioTechnology Institute, University of Minnesota, MN, USA

## Abstract

In soils halogens (fluorine, chlorine, bromine, iodine) are cycled through the transformation of inorganic halides into organohalogen compounds and vice versa. There is evidence that these reactions are microbially driven but the key enzymes and groups of microorganisms involved are largely unknown. Our aim was to uncover the diversity, abundance and distribution of genes encoding for halogenating and dehalogenating enzymes in a German forest soil by shotgun metagenomic sequencing. Metagenomic libraries of three soil horizons revealed the presence of genera known to be involved in halogenation and dehalogenation processes such as *Bradyrhizobium* or *Pseudomonas*. We detected a so far unknown diversity of genes encoding for (de)halogenating enzymes in the soil metagenome including specific and unspecific halogenases as well as metabolic and cometabolic dehalogenases. Genes for non-heme, no-metal chloroperoxidases and haloalkane dehalogenases were the most abundant halogenase and dehalogenase genes, respectively. The high diversity and abundance of (de)halogenating enzymes suggests a strong microbial contribution to natural halogen cycling. This was also confirmed in microcosm experiments in which we quantified the biotic formation of chloroform and bromoform. Knowledge on microorganisms and genes that catalyze (de)halogenation reactions is critical because they are highly relevant to industrial biotechnologies and bioremediation applications.

Halogenated organic compounds are diverse and widespread in nature. For a long time it was assumed that these compounds are only produced and released by anthropogenic sources^1^. Organohalogens like perchloroethene and trichloroethene are prominent groundwater pollutants due to their industrial use as dry cleaning and degreasing agents and their widespread accidental and deliberate release into the environment^2^. Volatile organohalogens (VOX) like chloromethane strongly influence atmospheric chemistry and thereby Earth’s climate by causing ozone depletion when released into the atmosphere^3^,^4^. Many organohalogens are of biological relevance e.g. in secondary metabolism. They are involved in various chemical defence mechanisms^5^, like the synthesis of the antibiotic pyrrolnitrin used in microbial antagonism by *Pseudomonas fluorescens*^6^. Furthermore, organohalogens, e.g. chloromethane, are metabolites involved in enzymatic lignin decomposition by fungi^7^,^8^,^9^. To date, over 5000 naturally occurring organohalogen compounds have been identified^10^. Abiotic sources of organohalogens in the environment are e.g. volcanic activities^11^ and biomass burning^12^,^13^. In soils organohalogens are produced during the abiotic oxidation of organic matter by Fe(III)^14^. The release of organohalogens, especially of VOX, has been demonstrated for various environments such as hypersaline lakes^15^,^16^, freshwater wetlands^17^, marine environments^18^,^19^ and soils^14^,^20^,^21^,^22^,^23^. The occurrence of a natural halogen cycling in soils was demonstrated in several studies, which mainly focused on the natural cycling of chlorine^24^,^25^,^26^. The turnover of chlorine in soil, namely the formation and decomposition of organic chlorine is due to both biotic and abiotic reactions^27^,^28^. However, it was shown that the natural chlorination processes in soils are primarily biotic^29^,^30^. Furthermore, several studies provided evidence for biotic dehalogenation potential in soils and their important environmental implications for contaminant removal^31^,^32^,^33^. Biotic halogenation and dehalogenation reactions are catalyzed by enzymes. A major group of halogenating enzymes are the haloperoxidases which unspecifically halogenate organic matter using hydrogen peroxide and a halogen ion (Cl^−^, Br^−^, I^−^) as substrate^34^,^35^,^36^,^37^. Based on their cofactors they can be classified into heme-dependent haloperoxidases^38^ and vanadium-dependent haloperoxidases^39^. Perhydrolases, or non-heme, no-metal haloperoxidases also require hydrogen peroxide and catalyze unspecific halogenation reactions but do not contain any metal cofactors^36^. Beside the haloperoxidases also halogenases with specific and regioselective halogenation reaction mechanisms exist. Flavin-dependent halogenases are involved in bacterial secondary metabolism, e.g. antibiotic syntheses^40^. Another class of specific halogenases are the alpha-ketoglutarate-dependent halogenases^41^. One known halogenase, a bacterial fluorinase, is able to fluorinate S-adenosyl-L-methionine via a nucleophilic mechanism^42^. Furthermore, methyltransferases of plants, fungi and algae^43^ are known to form halomethanes. Since organohalogen compounds are prominent environmental pollutants, their biotic degradation has been studied intensely in the past decades and a variety of different dehalogenation pathways including hydrolytic dehalogenation, dehydrohalogenation, thiolytic dehalogenation and intramolecular substitution have been described^36^,^44^. Dehalogenation of halomethanes by methyltransfer was described for bacterial methyltransferases^45^,^46^. Microorganisms can use organohalogens either as carbon source (metabolic degradation)^31^ or they are co-metabolically degraded during the degradation of primary substrates such as methane^47^. Metabolic and cometabolic degradation of organohalogens are possible under oxic and anoxic conditions^31^,^48^. Organohalogens can even be used as terminal electron acceptor in a metabolism called organohalide respiration^49^. Numerous pathways and enzymes involved in biotic halogenation and dehalogenation reactions have been identified. But so far little is known about the natural diversity and abundance of the different groups of halogenating and dehalogenating enzymes. It is further not well understood which genes and microorganisms are the main contributors to biotic halogen cycling^27^,^28^. Natural halogenation in soils is widespread and not only restricted to forest soils. It also occurs in grasslands and agricultural soils and the microbial chlorination and dechlorination of soil organic matter seems to be an ubiquitous phenomenon^50^. Knowledge on the microbial potential for halogenation and dehalogenation reactions in soils is important, since soils act as important sources of volatile organohalogens (e.g. CHCl_3_^51^), as well as sinks for natural and anthropogenic organohalogen compounds^32^. Here we combined geochemical analyses with microcosm experiments and shotgun metagenomics to unravel the natural diversity and relative abundance of genes encoding for halogenating and dehalogenating enzymes in a forest soil.

## Material and Methods

### Sampling

**Figure 1:**
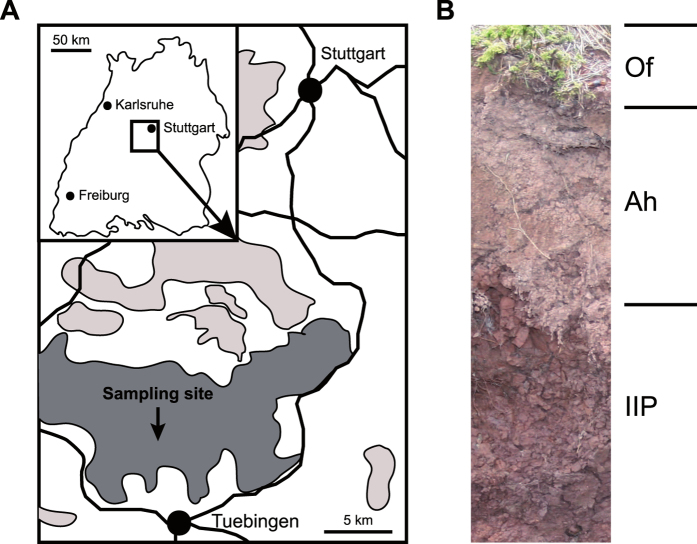
(A) Map of southern Germany and the location of the sampling site within the Schoenbuch wildlife park. Areas shaded in light grey represent forest areas, whereas the area shaded in dark grey represents the Schoenbuch wildlife park territory. **(B)** Soil depth profile at the sampling site with the two topsoil horizons (Of and Ah) and one subsoil horizon (IIP). The map was created with Adobe Illustrator CC (URL: http://www.adobe.com/products/illustrator.html).

The sampling site (N 48°30′24″, E 9°02′29″, WGS) is located in the Schoenbuch wildlife park, a forest close to Tuebingen in Southwest Germany (Fig. 1A). The forest area is predominated by beech with populations of oak, spruce and bald cypress. The soil has been qualified as vertic cambisol (WRB^52^). Three soil horizons were distinguished according to the German Soil Classification^53^: Of-horizon (1–0 cm), Ah-horizon (0–15 cm) and IIP-horizon (15–40 cm) (Fig. 1B).

At the sampling site two replicate soil profiles were sampled within a distance of 2 m from each other. Bulk soil samples for each profile were collected from the three distinguishable horizons of the top 40 cm, homogenized and stored at −80 °C for genetic analysis. For biogeochemical analysis bulk samples of the two soil profiles were mixed, homogenized and stored at 4 °C. Samples were taken in October 2013.

### Geochemical analysis

For water content determination, fresh soil samples were weighed and subsequently dried at 105 °C until weight stability. pH was measured in a suspension of 10 g air dried soil in 25 mL of a 0.01 M CaCl_2_-solution. For determination of leachable chloride and leachable organic carbon, 10 g of soil were mixed with 100 mL deionized water and shaken at 150 rpm for 24 h on a rotary shaker. Samples were centrifuged for 5 minutes at 4000 × *g* and filtered through a 0.45 μm pore size cellulose ester filter (Millex HA filter, EMD Millipore Corporation, USA). Dissolved organic carbon was measured with a High TOC Elementar system (Elementar Analysensysteme GmbH, Hanau, Germany) and chloride was determined by ion chromatography (Dionex DX 120, Thermo Scientific, Sunnyvale, CA, USA). For total organic carbon analysis soil samples were dried at 40 °C and sieved (2 mm mesh) to exclude large roots and stones. The organic carbon content was determined by heat combustion (1150 °C) and thermal conductivity analysis on a CNS element analyzer (Elementar Vario EL III, Elementar Analysensysteme GmbH, Hanau, Germany). Adsorbable organic halogen (AOX) content in the soil samples was determined according to the standard protocol (DIN EN ISO 9562) for soil leachates (DIN EN 12457-4) at the Laboratory for Environmental and Product Analytics (DEKRA GmbH, Halle, Germany).

### Detection of volatile organohalogen compounds

Microcosm experiments to quantify formation of volatile organohalogen compounds (VOX) in the soil horizons via GC-MS were set up in triplicates per soil horizon as follows: 3.5 g of native soil were incubated with 8.5 mL of sterile deionized water and incubated for 1 h at 30 °C in the dark prior to VOX quantification. Details on incubation conditions and GC-MS measurements have been published previously^15^.

### DNA extraction

Three different methods were applied to extract genomic DNA from the replicate soil samples. 10 g of soil were used for extraction with the PowerMax^®^ Soil DNA Isolation Kit (MoBio Laboratories, Carlsbad, CA, USA). Furthermore we applied a microwave-based extraction method^54^ with the following modifications: all steps were up-scaled for the extraction of DNA from 6 g of soil and DNA was precipitated by mixing the supernatant from the chloroform-isoamylalcohol-extraction with an equal amount of isopropanol followed by a 1 h incubation step at room temperature. The third DNA extraction protocol was based on a sodium-dodecyl-sulfate method combined with freeze-thawing, protein digestion and chloroform-isoamylalcohol extraction^55^. Since the DNA extracts of the latter two methods were still of brownish color, DNA was further purified by agarose gel electrophoresis using 0.7% agarose gels. High molecular weight DNA bands were excised from the agarose gels and subsequently extracted and purified using the Wizard^®^ SV Gel and PCR Clean-Up System (Promega, Madison WI, USA). DNA extracts were stored at −20 °C until further processing. Prior to sequencing DNA extracts derived from the replicate soil samples were pooled in equimolar quantities per sample. Quality and molecular weight of the genomic DNA extracts were confirmed by agarose gelelectrophoresis. 260/280 nm absorbance ratio as a measure of DNA purity was determined with a NanoDrop^®^ ND-1000 Spectrophotometer (Thermo Fisher Scientific, Wilmington, DE, USA).

### Metagenomic sequencing

For each of the duplicate soil samples a shotgun library was created. Shotgun library preparation and metagenome sequencing was performed at IMGM Laboratories GmbH (Martinsried, Germany). The shotgun library was prepared using the Nextera^®^ XT Sample Preparation technology (Illumina, San Diego, CA, USA). The libraries were size selected using Agencourt^®^ AMPure^®^ XP beads (Beckman Coulter, Pasadena, CA, USA) with a bead to DNA ratio of 0.6 to 1 (v/v). Quality and purity of the libraries has been analyzed with the High Sensitivity DNA Analysis Kit (Agilent Technologies, Santa Clara, CA, USA) on a 2100 Bioanalyzer (Agilent Technologies, Santa Clara, CA, USA). Prior to library normalization the libraries were quantified using the Quant-iT™ PicoGreen^®^ dsDNA assay kit (Invitrogen, Eugen, OR, USA). Sequencing was performed on an Illumina Miseq^®^ sequencing system (Illumina, San Diego, CA, USA) with the MiSeq Reagent Kit v3 (Illumina, San Diego, CA, USA) resulting in a read length of 2 × 300 bp. Signal processing, de-multiplexing and trimming of adapter sequences were performed using the MiSeq^®^ Reporter Software v. 2.3.32 (Illumina, San Diego, CA, USA).

### Quality processing, sequence alignment, taxonomic and functional analysis

Quality processing was performed using the Metagenomic RAST server^56^. Quality processing included trimming of low quality bases with the SolexaQA software package^57^ and a phred score of 30 as the lowest cutoff for a high quality base. Subsequently artificial duplicate reads produced by sequencing artifacts^58^ were removed with a k-mer based approach. For annotation unassembled reads were aligned against the non-redundant NCBI Reference Sequence (RefSeq) protein database using the program DIAMOND^59^ with a minimum percentage identity cutoff of 70% for protein sequences and an e-value cutoff of 1 × 10^−10^. The top 50 hits matching the cutoff criteria for each read were retained for further analysis. Phylogenetic analysis was performed in MEGAN 5^60^ using the Lowest Common Ancestor (LCA) algorithm only considering hits within the top 1% of the best bit score and a minimum bit score of 50. The LCA algorithm assigns species-specific sequences to specific taxa. Sequences that are conserved among different species (e.g. as consequence of horizontal gene transfer) will only be assigned to taxa of higher rank^60^. Nonetheless, it is very difficult to directly prove that a given (de)halogenase gene appears in a specific microbial taxon. Whenever we mention a specific species name in the results and discussion we refer to bacteria, archaea, or eukarya that contain a (de)halogenase gene closely related to the (de)halogenase gene of the respective species. Functional analysis using MEGAN 5 was based on the KEGG database and classification^61^. Each of the top 50 RefSeq hits for a read was mapped to a KEGG orthology (KO) group by identifying the best hit for a reference sequence for which a KO assignment is known. For the final assignment of a read to a KO group the KO assignment with the highest bitscore (best hit) of the assignments for the top 50 hits per read was selected. Reads related to genes of halogenating and dehalogenating enzymes were identified by analyzing reads assigned to KO groups for halogenating and dehalogenating enzymes. Since KO groups do not cover all halogenating and dehalogenating enzymes, we additionally aligned all reads with no hits to KEGG against specific databases for halogenating and dehalogenating enzymes using DIAMOND and the same cutoffs as for the RefSeq-annotation. Specific databases were created by searching the protein databases UniProt^62^ and Peroxibase^63^ for halogenating and dehalogenating enzymes. The specific databases include only enzymes of organisms for which halogenation or dehalogenation activity had been experimentally proven and published. “Putative enzymes” were not considered. KEGG hits and specific database hits were combined for relative abundance calculation. Abundances of functional genes were normalized to the total number of reads in the corresponding library and expressed as hits per million metagenomic reads. Abundance calculations for taxonomic groups were expressed relative to the number of all reads with a taxonomic assignment in the metagenomic library. Sequencing reads of the 12 metagenomic libraries are publically available via the MG-RAST metagenomic analysis server under project ID number 11442.

### Statistical analysis

Statistical comparison of the abundance of functional features between the soil horizons was performed using STAMP^64^ applying Analysis of Variance (ANOVA) as statistical test combined with the Tukey-Kramer method as post-hoc test. If the p-value for the 95% confidence interval was below 0.05, differences were considered significant. In statistical analyses each soil horizon included the data for the forward and reverse reads of the duplicate metagenome libraries (n = 4). To visualize differences in gene abundance between the soil horizons row z-sores were calculated in R^65^. Row z-scores represent the numbers of standard deviations a value differs from the mean.

## Results and Discussion

### Geochemical potential for natural halogenation and dehalogenation reactions

Total organic carbon and water-leachable and therefore potentially bioavailable carbon were highest in the Of-horizon with 301 g/kg dry soil and 619.4 mg/kg dry soil, respectively (Table 1). Water-leachable AOX was highest in the Of-horizon with 0.48 mg/kg dry soil and decreased with soil depth. The performed AOX measurements only provide information on the water-leachable AOX-compounds. However, it is important to note that also the non-soluble fraction of the soil matrix contains halogenated organic compounds.

Soluble AOX gradients correlated with organic carbon and chloride gradients in the Schoenbuch soil. Especially the Of-horizon in the Schoenbuch forest was characterized by a high content of weathering plant material. Transformation of inorganic chloride during humification of plant material leads to the rapid formation of stable and less volatile aromatic organohalogen compounds^66^. Our results support previous findings in the way that the presence of both organic carbon and halide ions stimulate natural halogenation and dehalogenation reactions in soil and that elevated organic matter contents accelerates chlorination rates^67^.

### Formation of volatile organohalogens in soil microcosm experiments

**Figure 2:**
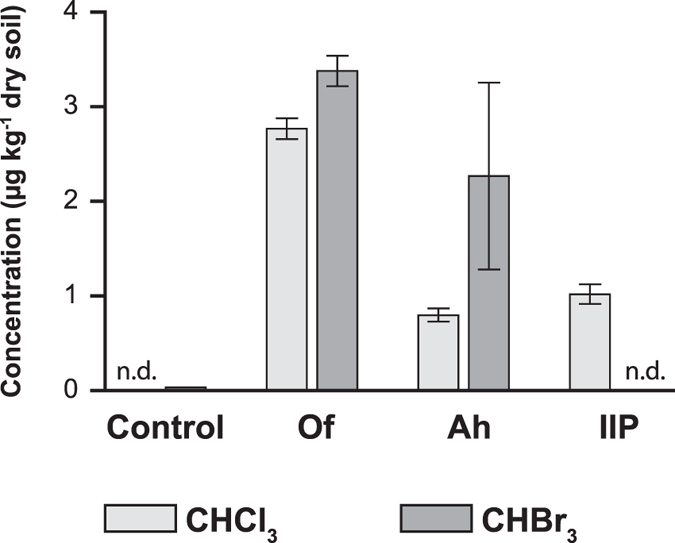
Emissions of chloroform (CHCl_3_) and bromoform (CHBr_3_) from microcosms with Schoenbuch forest soil from the three horizons Of, Ah and IIP after 1 h of incubation. The control contained only sterile incubation solution (no soil). Error bars indicate the standard deviation of three independent measurements. n.d. = not detected.

Besides AOX we followed the natural formation of volatile organohalogen compounds (VOX) in soil from the Schoenbuch forest. We observed the formation of chloroform (CHCl_3_) and bromoform (CHBr_3_) in soil microcosms after 1 h of incubation (Fig. 2). Highest VOX concentrations were observed for the Of-horizon with 2.8 ± 0.2 and 3.4 ± 0.3 μg/kg dry soil for chloroform and bromoform, respectively.

Soils are a known natural source of chloroform^22^,^68^. Furthermore it was demonstrated that in presence of inorganic bromide the formation of bromoform in soil is detectable^69^. In all three soil horizons of the Schoenbuch forest bromide could not be detected by ion chromatography. However, the formation of bromoform was observed in the OF- and Ah-horizon suggesting the presence of sufficient amounts of bromide for microbial bromoform formation. Especially soils with high organic carbon content due to decaying plant material and a rich humic layer were prominent sources for chloroform^68^. This was confirmed for the Schoenbuch forest soil, from which the emissions of trihalomethanes were highest in the organic rich Of-horizon. A recent study on chloroform formation from humic substances in soils using stable isotope analysis suggested microbial formation via extracellular chloroperoxidases as potential source of VOX formation^70^. The predominance of microbial chlorination over abiotic chlorination reactions in forest soils was demonstrated by a clear temperature sensitivity of the observed chlorination reactions^29^ and the significantly lower chlorination of organic matter in autoclaved and/or gamma sterilized soils^30^. Also microcosm studies on microbial dehalogenation revealed that both, anaerobic dehalogenation^32^ and aerobic dehalogenation^33^ of organohalogen compounds by microorganisms prevailed over abiotic reactions in the investigated soils. Both microbial halogenation and dehalogenation reactions in soils contribute to the natural halogen cycling, but so far the diversity and abundance of the involved microorganisms and enzymes have not been studied in great detail^27^,^28^. Since soluble and volatile organohalogen compounds were detectable in incubation experiments with Schoenbuch forest soil we used a shotgun metagenomic sequencing approach to investigate the genetic potential for microbial halogenation and dehalogenation reactions.

### General information on the Schoenbuch metagenome

**Figure 3:**
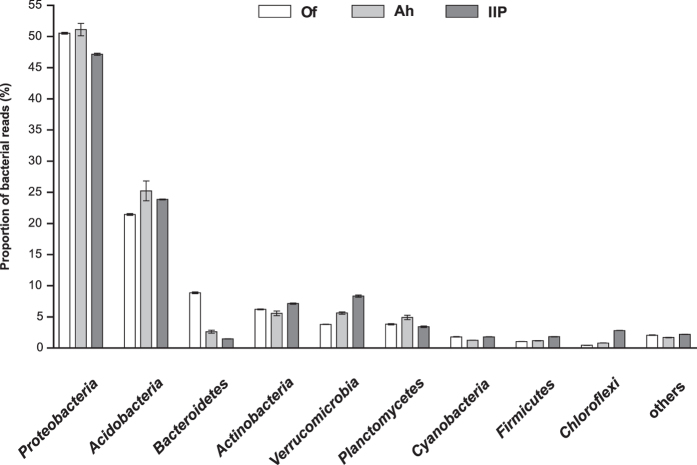
Mean proportion of bacterial phyla in the three soil horizons Of, Ah and IIP. Relative percentages were calculated for all reads assigned to the domain Bacteria. Error bars indicate the standard deviation of the mean for the forward and reverse metagenomic read libraries of duplicate samples for each soil horizon (n = 4).

Metagenomic sequencing of two replicate samples per soil horizon resulted in a total of 38.8 million reads with a read length of 300 bp. After quality processing a total of 36.2 million high quality reads were used for taxonomic and functional analysis. Detailed sequencing statistics for the metagenome libraries of the duplicate soil samples are given in Table S1. Taxonomic classification was possible for 20.4% of the metagenomic reads, whereas functional annotation was possible for 8.5% of the reads. Since our study focuses on the microbial halogen cycle only sequences related to Bacteria, Archaea or Fungi were considered in our analysis. Of the reads that could be taxonomically assigned 99.5% were related to Bacteria, whereas 0.1% and 0.4% were related to Archaea and Fungi, respectively. The higher relative abundance of Bacteria over Archaea was confirmed by quantifying 16S rRNA gene copy numbers of both domains by qPCR (results shown in Table S2). 16S rRNA gene copy numbers in the three soil horizons were approximately three orders of magnitude higher for bacterial 16S rRNA genes compared to archaeal 16S rRNA genes. However, 16S rRNA gene copy numbers were not corrected for ribosomal rRNA gene operon numbers. Strong predominance of bacterial over archaeal reads in soil metagenomic libraries has also been demonstrated in a cross-metagenomic survey of 16 different soil samples^71^ and metagenomics analyses of permafrost soils^72^. Bacterial reads in the Schoenbuch forest soil metagenome were mainly related to the *Proteobacteria* (47.2–50.5%) and *Acidobacteria* (21.4–24.0%) (Fig. 3). Further, reads affiliated to *Bacteroidetes*, *Actinobacteria* and *Verrucomicrobia* constituted considerable fractions of all bacterial reads.

The dominant bacterial phyla in the Schoenbuch forest soil are typical members of soil microbial communities and represented the majority of the bacterial reads in metagenomes of e.g. desert and forest soils^71^, tallgrass prairie soils^73^ and a boreal forest soil^74^. Functional metagenomic reads were mainly associated with the KEGG subsystem metabolism (43.9–45.2%) or could not directly be grouped within one of the KEGG subsystems (29.2–29.9%) (Figure S1).

### Identification of microorganisms and enzymes possibly involved in natural halogen cycling in Schoenbuch forest soil

We screened the metagenome for microorganisms that are known to possess genes encoding for enzymes that perform halogenation or dehalogenation reactions or for which halogenation and dehalogenation reactions have been confirmed by experimental approaches (Table 2). Relative abundances of these taxa were calculated on the genus rank, since taxonomic classification at the species or strain level is not reliable for short metagenomic reads.

*Bradyrhizobium* and *Burkholderia* were the most abundant genera possessing genes for both, halogenating and dehalogenating enzymes. With the exception of eleven fungal genera and one archaeal genus all other genera belonged to the Bacteria indicating that halogen cycling might be mainly bacteria driven in the investigated forest soil. Most taxa in Table 2 are facultative aerobic microorganisms suggesting the prevalence of aerobic halogenation and dehalogenation pathways. Anaerobic bacteria known for reductive dehalogenation such as *Dehalococcoides* or *Dehalobacter* were less abundant, probably because the top 40 cm of the Schoenbuch forest soil were mainly oxic. Nonetheless, anoxic microsites in water filled micropores could sustain growth and activity of reductively dehalogenating microorganisms even in primarily oxic soil horizons.

**Figure 4:**
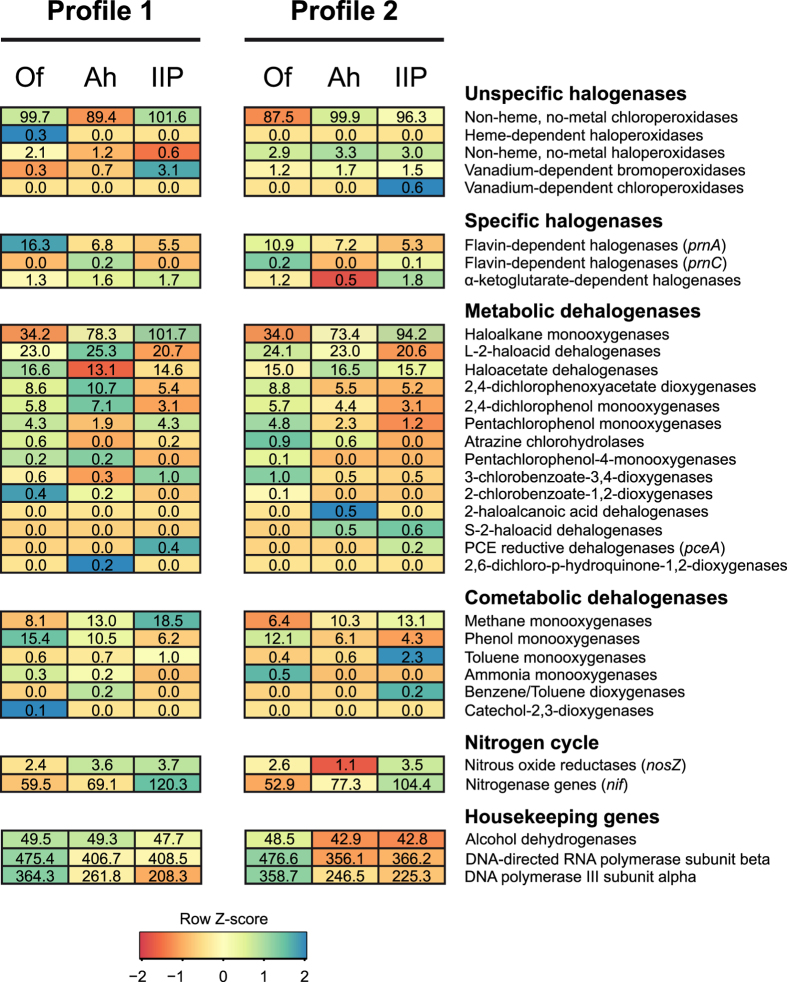
Heatmap summarizing the relative abundance of reads annotated as halogenase and dehalogenase genes in the metagenomic libraries of the replicate soil samples. The relative abundance of genes of the nitrogen cycle and of selected housekeeping genes is given as reference. Functional assignments are based on 70% amino acid sequence identity and an e-value of 1 × 10^−10^. The color code represents the row z-score, the number of standard deviations a value differs from the mean. Numeric values within the heatmap represent the relative abundance in hits per million metagenomic reads. In samples with a relative abundance of 0.0 no reads for the corresponding enzyme were found.

In order to assess the genetic potential for microbial halogenation and dehalogenation reactions in the Schoenbuch forest soil, we tried to identify reads that encode for halogenating and dehalogenating enzymes. Their relative abundances in the metagenomic libraries of the duplicate samples of each soil horizon are displayed in Fig. 4. The applied metagenomic approach revealed a high genetic diversity for halogenating and dehalogenating enzymes covering a variety of different halogenation and dehalogenation mechanisms. Most retrieved halogenase genes encoded for enzymes with oxidative halogenation mechanisms. Also Vaillancourt *et al*. described that oxidative halogenation pathways predominate in many ecosystems^37^. Furthermore experiments on the chlorination of organic matter in forest soils suggested oxygen-dependent enzymes driving the biotic chlorination in soils^29^. For dehalogenating enzymes a variety of oxidative and reductive dehalogenation reactions are known. The majority of the dehalogenase genes we found in the Schoenbuch soil metagenome were related to hydrolytic or oxidative dehalogenases^31^. The only reductive dehalogenase genes we identified were related to a *pceA* gene encoding for a reductive dehalogenase that catalyses the dechlorination of perchloroethene and trichloroethene^75^. The relative abundances of the most abundant halogenase and dehalogenase genes were in the same order of magnitude as functional genes involved in microbial nitrogen cycling (*nosZ*, *nif*-genes) or housekeeping genes such as e.g. DNA or RNA polymerases. The fact that halogenase and dehalogenase genes occurred at relative abundances similar to essential soil microbial community functions emphasizes the importance of these enzymes for (de)halogenation reactions in forest soils and suggests a major role of bacteria in the cycling of halogens in soils.

The heatmap in Fig. 4 shows that the relative abundances of halogenase and dehalogenase genes in the two replicate metagenomic libraries were in the same order of magnitude and follow the same trends with soil depth. Therefore, we combined the data of both libraries for further analysis. The variance between the replicate libraries is then reflected by the given standard deviation.

We verified the relative abundances of selected halogenase, dehalogenase and reference genes involved in nitrogen cycling in the different soils horizons of the metagenome data set by qPCR. For the four selected genes (the haloalkane dehalogenase gene *dhaA* of *Mycobacterium smegmatis*, the flavin-dependent halogenase gene *prnA* of *Pseudomonas fluorescens, nosZ*, and, *nifH*) qPCR results confirmed the observed trends in relative read abundances across the different soil horizons (Table S3).

**Figure 5:**
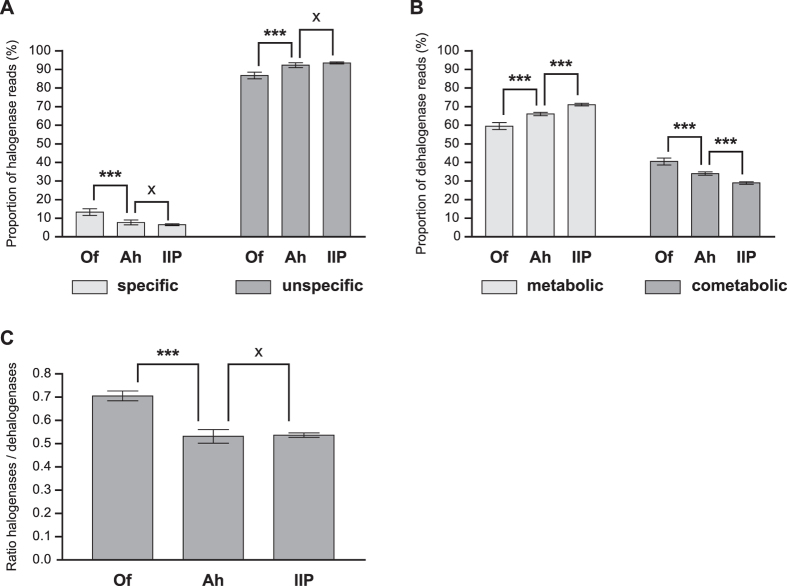
Proportion of reads for specific and unspecific halogenases **(A)** or metabolic and cometabolic dehalogenases **(B)** in the three soil horizons. **(C)** Ratio of halogenase and dehalogenase gene abundance in the soil horizons. A ratio of 1 represents an equal abundance and a ratio below 1 a higher abundance of dehalogenase genes. Horizons were compared using ANOVA and statistical significant differences are marked by asterisks (*p < 0.05, **p < 0.01, ***p < 0.001). x indicates no significant differences (*p* > 0.05). Only comparisons for adjacent soil horizons are shown.

Of major interest with respect to halogenating enzymes is the proportion of genes encoding for either specific or unspecific halogenases. Genes for unspecific halogenases represented 86.7–93.5% of the total halogenase reads whereas genes for specific halogenases represented 6.5–13.3% (Fig. 5A). Unspecific halogenases increased significantly with sediment depth although differences between the Ah- and IIP-horizon were not significant. All unspecific halogenases were haloperoxidases. The higher proportion of unspecific halogenases in the deeper soil horizons might be related to their ability of reducing hydrogen peroxide to water. The rhizosphere at the sampling site was located at the intersection of the Ah- and IIP-horizon. Also the high abundance of nitrogen fixation genes (*nif*-genes) locates the rhizosphere near the IIP-horizon (Fig. 4). Haloperoxidases could be used by microorganisms as defence against oxidative stress induced by reactive oxygen species released by plants to antagonize pathogens and rhizosphere infections^76^. Specific halogenases such as flavin-dependent halogenases are involved in secondary metabolism, e.g. antibiotic synthesis^40^. 16S rRNA gene copy numbers and organic carbon content were highest in the Of-horizon suggesting that microbial competition and the necessity for production of antimicrobial agents might be high in this soil layer. This might also be a potential explanation for the high proportion of specific halogenase genes in the Of-horizon. The proportion of genes for metabolic dehalogenases significantly increased with soil depth. Metabolic dehalogenases constituted the major fraction of all dehalogenase assigned reads (59.5–71.7%), while genes encoding for cometabolic dehalogenases were less abundant (28.9–40.5%) (Fig. 5B). Many metabolic and cometabolic dehalogenases have a broad substrate specificity, e.g. the methane monooxygenases or haloalkane dehalogenases^31^.

Microorganisms using organohalogens as carbon source or electron acceptor are therefore not necessarily restricted by the availability of their primary substrate for their dehalogenating enzymes. This makes correlations between the abundance of dehalogenase genes and specific organohalogens formed during microbial halogenation reactions difficult. The relative proportion of cometabolic dehalogenases was highest in the Of-horizon. These enzymes utilize non-halogenated organic compounds as substrate and since the Of-horizon had the highest content of organic carbon, this might explain that organisms possessing monooxygenases or dioxygenases are abundant in this horizon, where they can utilize the available aromatic compounds, e.g. phenolic breakdown products of lignin degradation^77^. The ratio of halogenase to dehalogenase genes (Fig. 5C) revealed a higher abundance of dehalogenase genes in all soil horizons, whereas the ratio was closest to 1 in the Of-horizon (0.71). The observed ratio only displays the genetic potential for enzymatic halogenation or dehalogenation. Since gene expression and protein synthesis are dependent on many factors and differ strongly between different genes the relative abundance of functional genes in metagenomic datasets is no indicator of the importance of a certain function or activity in a given sample. However, since we quantified the net release of chloroform in all laboratory soil microcosms, chloroform formation must have been higher than chloroform degradation in all soil horizons.

**Figure 6:**
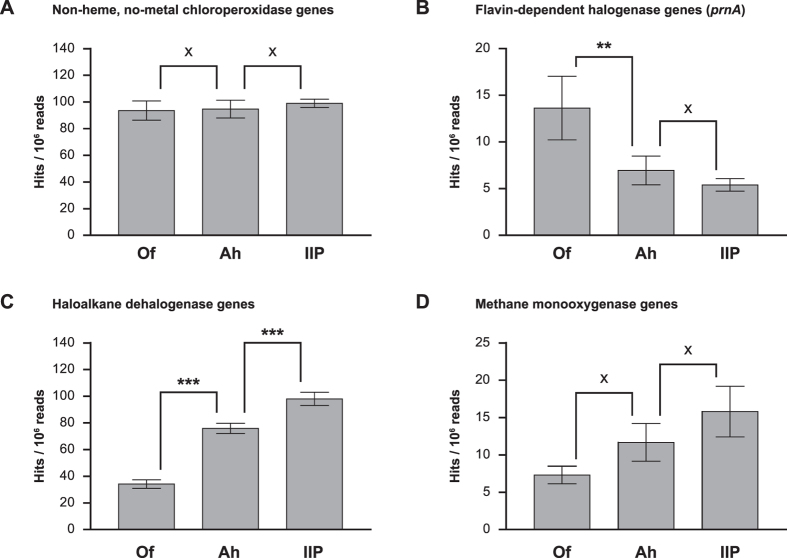
Abundance in hits per million metagenomic reads of non-heme, no metal chloroperoxidase genes **(A)**, flavin-dependent halogenase genes (*prnA*) **(B)**, haloalkane dehalogenase genes **(C)** and methane monooxygenase genes **(D)**. The four enzymes are the most abundant representatives of unspecific and specific halogenases and metabolic and cometabolic dehalogenases, respectively. Horizons were compared using ANOVA and statistical significant differences are marked by asterisks (*p < 0.05, **p < 0.01, ***p < 0.001). x indicates no significant differences (*p* > 0.05). Only comparisons for adjacent soil horizons are displayed.

For each halogenase and dehalogenase subgroup as classified in Fig. 4 we further investigated the distribution of the most abundant subgroup within the soil profiles. The most abundant unspecific dehalogenases were the non-heme, no metal chloroperoxidases (Fig. 6A) with 93.6–99.0 hits per million reads. No significant differences in abundance between the three soil horizons were detected. In general chloroperoxidases oxidize halides in the presence of hydrogen peroxide to the corresponding hypohalous acid, responsible for the unspecific halogenation of electron-rich organic matter^78^. The non-heme, no metal chloroperoxidases also use hydrogen peroxide for their halogenation mechanism^79^. These enzymes are also referred to as perhydrolases and show like the heme- or vanadium containing chloroperoxidases no substrate specificity^36^. Therefore, a role in synthesis of specific compounds can be excluded. It was hypothesized that chloroperoxidases might be involved in microbial antagonism through the production of reactive chlorine species as antimicrobial agents^80^. As mentioned above the ability to reduce hydrogen peroxide to water suggests a role in oxidative stress response by microorganisms associated with the rhizosphere of plants. Many plant-associated organisms, e.g. *Sinorhizobium meliloti*^81^, possess non-heme, no metal chloroperoxidases^76^. Due to their reaction mechanism it is likely that chloroperoxidases are involved in the formation of chloroform in soils^51^,^70^. The high abundance of genes for chloroperoxidases in the Schoenbuch forest soil might be a possible explanation for the observed formation of chloroform in our microcosm experiments. Since chloroperoxidases can also use bromide^37^, these enyzmes might also play a role in the formation of bromoform.

Halogenase genes such as the flavin-dependent halogenase prnA were the most abundant specific halogenases in the dataset. The Of-horizon revealed a significantly higher abundance of *prnA* genes (13.6 hits per million metagenomic reads) compared to the Ah and IIP-horizon (Fig. 6B). The *prnA* gene encodes for a tryptophan-7 halogenase which is together with a second flavin-dependent halogenase (prnC) involved in the biosynthesis of the antifungal antibiotic pyrrolnitrin^40^. As discussed above the role of the PrnA enzyme in antibiotic synthesis might explain the higher abundance of the *prnA* gene in the Of-horizon. The carbon content, bacterial and archaeal cell numbers (as approximated by 16S rRNA gene copy numbers), and the abundance of fungal metagenomic reads were highest in the Of-horizon. Therefore this soil horizon might constitute a microbial habitat in which the genetic potential for the production of antimicrobials could provide a competitive advantage which might explain the increased abundance of genes involved in synthesis of an antifungal antibiotic such as pyrrolnitrin.

The abundance of haloalkane dehalogenase genes was highest in the IIP-horizon (98.0 hits per million reads) and decreased significantly in the Ah- and Of-horizon (*p* < 0.001) (Fig. 6C). Haloalkane dehalogenases are hydrolytic dehalogenases and have a broad substrate spectrum including various chlorinated and brominated aliphatic compounds^82^. The IIP-horizon was characterized by the lowest soluble AOX content which might be indicative of active dehalogenation mechanisms, also suggested by the high genetic potential for metabolic dehalogenation reactions in the IIP-horizon. The lower emissions of chloroform and the absence of bromoform formation in soil from the IIP-horizon could be due to an elevated activity of dehalogenating enzymes involved in the degradation of chloroform and bromoform.

Methane monooxygenase genes represented the most prominent genes among the cometabolic dehalogenases (Fig. 6D). Their abundance was highest in the IIP-horizon (15.8 hits per million reads). Comparison between directly adjacent horizons revealed no significant differences in abundance but the difference in abundance between the Oh- and IIP-horizon was significant (*p* < 0.01). Methanotrophs primarily use methane monooxygenases to catalyze the oxidation of methane to methanol but the enzyme can also oxidize a wide range of alkanes and alkenes^83^. Methane monooxygenases are also known for cometabolic oxidation of halogenated alkenes^84^ and alkanes such as chloroform^85^. The high abundance of methane monooxygenase genes in the IIP-horizon suggests the occurrence of high numbers of methanotrophic bacteria in this soil horizon. Following rainfalls parts of the IIP-horizon might quickly turn anoxic which could promote methanogenesis and the activity of methanotrophs in oxic zones since activities of methanotrophic bacteria and methanogenic archaea in soil are known to be correlated^86^.

## Summary and Outlook

The microbial mechanisms driving the halogen cycle in soils are mainly unknown. Therefore knowledge on formation and degradation processes is important to evaluate the role of soils as sinks or sources of organohalogen compounds^87^. The metagenomic survey conducted in this study revealed a tremendous diversity and high abundance of genes encoding for halogenating and dehalogenating enzymes in the investigated soil. Although we did not analyze gene expression or enzyme activity we could show that the studied forest soil harbours the genetic potential for specific and unspecific halogenation as well as metabolic and cometabolic dehalogenation activities with a clear predominance of oxidative bacterial reaction pathways. However, the relative contribution of the different enzymatic groups to the overall cycling of organic and inorganic halogens in the Schoenbuch requires further study. Here we demonstrated that metagenomics allows for the identification of the diversity and relative abundance of enzymatic halogenating and dehalogenating reaction mechanisms in soils that build the basis for further investigation of microbial halogen cycling. Since halogenating and dehalogenating enzymes use different reaction mechanisms for the (de)halogenation of organic matter the contribution of individual enzymatic mechanisms to overall halogen cycling should be further elucidated by stable chlorine isotope fractionation as recently demonstrated^70^,^88^. The combination of omics approaches, laboratory microcosm experiments, and stable isotope analysis constitutes a powerful set of tools to further investigate the microbial contribution to natural halogenation and dehalogenation reactions in soils.

## Additional Information

**How to cite this article**: Weigold, P. *et al*. A metagenomic-based survey of microbial (de)halogenation potential in a German forest soil. *Sci. Rep*. **6**, 28958; doi: 10.1038/srep28958 (2016).

## Supplementary Material

Supplementary Information

## Figures and Tables

**Table 1:** Physical and chemical properties in the three soil horizons Of, Ah and IIP of the Schoenbuch forest.

Horizon	Water content (%)	pH	TOC (g/kg)^a^	Leachable OC (mg/kg)^a^	Leachable AOX (mg/kg)^a^	Leachable Cl^−^ (mg/kg)^a^
Of	50.3	5.9	301	619.4	0.48	24.5
Ah	27.7	5.1	33	486.6	0.29	13.4
IIP	24.8	5.4	7	233.3	0.15	9.1

TOC: Total organic carbon.

OC: organic carbon.

^a^per kg dry soil

**Table 2:** Mean abundance of taxa known to possess enzymes for biotic halogenation or dehalogenation reactions.

Genus	Group	Halo	Dehalo	Relative abundance (%)		
				Of	Ah	IIP
*Bradyrhizobium*	Bacteria	x	x	10.381	12.549	9.038
Candidatus *Solibacter*	Bacteria	x		3.673	4.872	5.604
*Sphingomonas*	Bacteria		x	1.597	0.501	0.355
*Burkholderia*	Bacteria	x	x	1.499	1.505	1.490
*Mycobacterium*	Bacteria	x	x	1.002	1.122	0.742
*Mesorhizobium*	Bacteria	x		0.778	1.066	0.860
*Pseudomonas*	Bacteria	x	x	0.574	0.469	0.525
*Rhizobium*	Bacteria		x	0.566	0.839	0.727
*Streptomyces*	Bacteria	x		0.497	0.616	0.906
*Rhodopseudomonas*	Bacteria	x	x	0.447	0.485	0.356
*Methylobacterium*	Bacteria		x	0.370	0.553	0.422
*Cupriavidus*	Bacteria	x		0.271	0.294	0.355
*Polaromonas*	Bacteria		x	0.240	0.149	0.166
*Nocardioides*	Bacteria		x	0.222	0.063	0.070
*Hyphomicrobium*	Bacteria		x	0.175	0.203	0.163
*Actinoplanes*	Bacteria	x		0.160	0.119	0.139
*Myxococcus*	Bacteria	x		0.137	0.131	0.185
*Ralstonia*	Bacteria		x	0.129	0.137	0.212
*Sinorhizobium*	Bacteria	x		0.114	0.218	0.180
*Rhodococcus*	Bacteria	x	x	0.109	0.117	0.171
*Geobacter*	Bacteria		x	0.101	0.182	0.377
*Rhodospirillum*	Bacteria		x	0.099	0.134	0.109
*Methylosinus*	Bacteria		x	0.094	0.179	0.121
*Desulfovibrio*	Bacteria		x	0.093	0.126	0.188
*Amycolatopsis*	Bacteria	x		0.093	0.114	0.165
*Xanthobacter*	Bacteria		x	0.081	0.111	0.090
*Nitrosomonas*	Bacteria		x	0.046	0.045	0.072
*Clostridium*	Bacteria	x		0.043	0.042	0.063
*Ancylobacter*	Bacteria		x	0.030	0.034	0.027
*Salinispora*	Bacteria	x		0.026	0.035	0.051
*Desulfuromonas*	Bacteria		x	0.022	0.036	0.070
*Oscillatoria*	Bacteria	x		0.019	0.028	0.040
*Desulfomonile*	Bacteria		x	0.019	0.044	0.076
*Nostoc*	Bacteria	x		0.018	0.024	0.041
*Methylococcus*	Bacteria		x	0.017	0.023	0.032
*Corynebacterium*	Bacteria		x	0.015	0.017	0.018
*Shewanella*	Bacteria		x	0.015	0.014	0.014
*Nonomuraea*	Bacteria	x		0.015	0.022	0.038
*Anabaena*	Bacteria	x		0.009	0.015	0.026
*Microscilla*	Bacteria	x		0.008	0.003	0.003
*Lactobacillus*	Bacteria	x		0.008	0.008	0.011
*Synechocystis*	Bacteria	x		0.008	0.011	0.018
*Leisingera*	Bacteria		x	0.008	0.010	0.010
*Psychroflexus*	Bacteria	x		0.006	0.002	0.001
*Lechevalieria*	Bacteria	x		0.006	0.008	0.013
*Dehalobacter*	Bacteria		x	0.005	0.004	0.005
*Desulfitobacterium*	Bacteria		x	0.004	0.004	0.006
*Lyngbya*	Bacteria	x		0.004	0.007	0.010
*Dehalococcoides*	Bacteria		x	0.004	0.004	0.010
*Gramella*	Bacteria	x		0.003	0.002	0.002
*Actinosynnema*	Bacteria	x		0.003	0.003	0.004
*Moraxella*	Bacteria		x	0.001	0.002	0.001
*Sulfurospirillum*	Bacteria		x	0.001	0.002	0.002
*Acetobacterium*	Bacteria		x	0.001	0.001	0.001
*Weissella*	Bacteria	x		0.000	0.000	0.001
*Pediococcus*	Bacteria	x		0.000	0.001	0.000
*Methanosarcina*	Archaea		x	0.007	0.008	0.016
*Aspergillus*	Fungi	x		0.011	0.004	0.003
*Laccaria*	Fungi	x		0.011	0.011	0.002
*Agaricus*	Fungi	x		0.007	0.007	0.003
*Batrachochytrium*	Fungi	x		0.007	0.003	0.002
*Coprinopsis*	Fungi	x		0.005	0.007	0.002
*Auricularia*	Fungi	x		0.005	0.002	0.001
*Thielavia*	Fungi	x		0.003	0.001	0.000
*Podospora*	Fungi	x		0.002	0.001	0.001
*Neurospora*	Fungi	x		0.001	0.000	0.000
*Magnaporthe*	Fungi	x		0.001	0.000	0.000
*Ustilago*	Fungi	x		0.001	0.001	0.000

Relative abundances are given on the genus level. Dehalo: Genus comprises species with genetic dehalogenation potential. Halo: Genus comprises species with genetic halogenation potential. Some genera contain species that possess the genetic potential to perform both halogenation and dehalogenation reactions.

## References

[b1] Gribble G. W. Naturally occurring organohalogen compounds - a survey. J. Nat. Prod. 55, 1353–1395 (1992). 10.1021/acs.jnatprod.3c00803 38375796

[b2] Bradley P. M. History and ecology of chloroethene biodegradation: A review. Bioremediation J. 7, 81–109 (2003).

[b3] Butler J. H. Atmospheric chemistry: Better budgets for methyl halides? Nature 403, 260–261 (2000). 10.1038/35002232 10681236

[b4] Read K. A. . Extensive halogen-mediated ozone destruction over the tropical Atlantic Ocean. Nature 453, 1232–1235 (2008). 18580948 10.1038/nature07035

[b5] Laus G. In Studies in Natural Products Chemistry (ed. Atta-ur-Rahman) 25, Part F, 757–809 (Elsevier, 2001).

[b6] Hammer P. E., Hill D. S., Lam S. T., Van Pée K. H. & Ligon J. M. Four genes from *Pseudomonas fluorescen*s that encode the biosynthesis of pyrrolnitrin. Appl. Environ. Microbiol. 63, 2147–2154 (1997). 9172332 10.1128/aem.63.6.2147-2154.1997 PMC168505

[b7] de Jong E., Field J. A. & de Bont J. A. M. Aryl alcohols in the physiology of ligninolytic fungi. FEMS Microbiol. Rev. 13, 153–187 (1994).

[b8] Harper D. B., McRoberts W. C. & Kennedy J. T. Comparison of the efficacies of chloromethane, methionine, and S-adenosylmethionine as methyl precursors in the biosynthesis of veratryl alcohol and related compounds in *Phanerochaete chrysosporium*. Appl. Environ. Microbiol. 62, 3366–3370 (1996). 16535404 10.1128/aem.62.9.3366-3370.1996 PMC1388942

[b9] Anke H. & Weber R. W. S. White-rots, chlorine and the environment – a tale of many twists. Mycologist 20, 83–89 (2006).

[b10] Gribble G. W. A recent survey of naturally occurring organohalogen compounds. Environ. Chem. 12, 396–405 (2015).

[b11] Jordan A., Harnisch J., Borchers R., Le Guern F. & Shinohara H. Volcanogenic halocarbons. Environ. Sci. Technol. 34, 1122–1124 (2000).

[b12] Rudolph J., Khedim A., Koppmann R. & Bonsang B. Field study of the emissions of methyl chloride and other halocarbons from biomass burning in Western Africa. J. Atmospheric Chem. 22, 67–80 (1995).

[b13] Andreae M. O. & Merlet P. Emission of trace gases and aerosols from biomass burning. Glob. Biogeochem. Cycles 15, 955–966 (2001).

[b14] Keppler F., Borchers R., Pracht J., Rheinberger S. & Schöler H. F. Natural formation of vinyl chloride in the terrestrial environment. Environ. Sci. Technol. 36, 2479–2483 (2002). 12075808 10.1021/es015611l

[b15] Ruecker A. . Predominance of biotic over abiotic formation of halogenated hydrocarbons in hypersaline sediments in Western Australia. Environ. Sci. Technol. 48, 9170–9178 (2014). 25073729 10.1021/es501810g

[b16] Weissflog L., Lange C. A., Pfennigsdorff A. & Kotte K. Sediments of salt lakes as a new source of volatile highly chlorinated C1/C2 hydrocarbons. Geophys. Res. Lett. 32, 1–4 (2005).

[b17] Hardacre C. J. & Heal M. R. Characterization of methyl bromide and methyl chloride fluxes at temperate freshwater wetlands. J. Geophys. Res. Atmospheres 118, 977–991 (2013).

[b18] Ballschmiter K. Pattern and sources of naturally produced organohalogens in the marine environment: biogenic formation of organohalogens. Chemosphere 52, 313–324 (2003). 12738255 10.1016/S0045-6535(03)00211-X

[b19] Carpenter L. J., Liss P. S. & Penkett S. A. Marine organohalogens in the atmosphere over the Atlantic and Southern Oceans. J. Geophys. Res. Atmospheres 108, 4256 (2003).

[b20] Albers C. N., Jacobsen O. S., Flores É. M. M., Pereira J. S. F. & Laier T. Spatial variation in natural formation of chloroform in the soils of four coniferous forests. Biogeochemistry 103, 317–334 (2011).

[b21] Haselmann K. F., Laturnus F., Svensmark B. & Grøn C. Formation of chloroform in spruce forest soil – results from laboratory incubation studies. Chemosphere 41, 1769–1774 (2000). 11057617 10.1016/s0045-6535(00)00044-8

[b22] Haselmann K., Laturnus F. & Grøn C. Formation of chloroform in soil. A year-round study at a Danish spruce forest Site. Water. Air. Soil Pollut. 139, 35–41 (2002).

[b23] Keppler F. . De novo formation of chloroethyne in soil. Environ. Sci. Technol. 40, 130–134 (2006). 16433342 10.1021/es0513279

[b24] Bastviken D. . Chloride retention in forest soil by microbial uptake and by natural chlorination of organic matter. Geochim. Cosmochim. Acta 71, 3182–3192 (2007).

[b25] Öberg G. & Sandén P. Retention of chloride in soil and cycling of organic matter-bound chlorine. Hydrol. Process. 19, 2123–2136 (2005).

[b26] Öberg G., Holm M., Sandén P., Svensson T. & Parikka M. The role of organic-matter-bound chlorine in the chlorine cycle: A case study of the Stubbetorp catchment, Sweden. Biogeochemistry 75, 241–269 (2005).

[b27] Öberg G. The natural chlorine cycle – fitting the scattered pieces. Appl. Microbiol. Biotechnol. 58, 565–581 (2002). 11956738 10.1007/s00253-001-0895-2

[b28] Clarke N. . The formation and fate of chlorinated organic substances in temperate and boreal forest soils. Environ. Sci. Pollut. Res. 16, 127–143 (2009). 10.1007/s11356-008-0090-4 19104865

[b29] Bastviken D., Svensson T., Karlsson S., Sandén P. & Öberg G. Temperature sensitivity indicates that chlorination of organic matter in forest soil is primarily biotic. Environ. Sci. Technol. 43, 3569–3573 (2009). 19544856 10.1021/es8035779

[b30] Rohlenová J. . Microbial chlorination of organic matter in forest soil: investigation using ^36^Cl-Chloride and its methodology. Environ. Sci. Technol. 43, 3652–3655 (2009). 19544868 10.1021/es803300f

[b31] Fetzner S. Bacterial dehalogenation. Appl. Microbiol. Biotechnol. 50, 633–657 (1998). 9891928 10.1007/s002530051346

[b32] Borch T., Ambus P., Laturnus F., Svensmark B. & Grøn C. Biodegradation of chlorinated solvents in a water unsaturated topsoil. Chemosphere 51, 143–152 (2003). 12586147 10.1016/S0045-6535(02)00851-2

[b33] Miller L. G. . Degradation of methyl bromide and methyl chloride in soil microcosms: Use of stable C isotope fractionation and stable isotope probing to identify reactions and the responsible microorganisms. Spec. Issue Microb. Geochem. 68, 3271–3283 (2004).

[b34] Blasiak L. C. & Drennan C. L. Structural perspective on enzymatic halogenation. Acc. Chem. Res. 42, 147–155 (2003). 18774824 10.1021/ar800088r PMC3980734

[b35] Butler A. & Sandy M. Mechanistic considerations of halogenating enzymes. Nature 460, 848–854 (2009). 19675645 10.1038/nature08303

[b36] van Pée K. H. & Unversucht S. Biological dehalogenation and halogenation reactions. Chemosphere 52, 299–312 (2003). 12738254 10.1016/S0045-6535(03)00204-2

[b37] Vaillancourt F. H., Yeh E., Vosburg D. A., Garneau-Tsodikova S. & Walsh C. T. Nature’s inventory of halogenation catalysts: oxidative strategies predominate. Chem. Rev. 106, 3364–3378 (2006). 16895332 10.1021/cr050313i

[b38] Hofrichter M., Ullrich R., Pecyna M. J., Liers C. & Lundell T. New and classic families of secreted fungal heme peroxidases. Appl. Microbiol. Biotechnol. 87, 871–897 (2010). 20495915 10.1007/s00253-010-2633-0

[b39] Butler A. Mechanistic considerations of the vanadium haloperoxidases. Coord. Chem. Rev. 187, 17–35 (1999).

[b40] Van Pée K. H. & Patallo E. Flavin-dependent halogenases involved in secondary metabolism in bacteria. Appl. Microbiol. Biotechnol. 70, 631–641 (2006). 16544142 10.1007/s00253-005-0232-2

[b41] Vaillancourt F. H., Yeh E., Vosburg D. A., O’Connor S. E. & Walsh C. T. Cryptic chlorination by a non-haem iron enzyme during cyclopropyl amino acid biosynthesis. Nature 436, 1191–1194 (2005). 16121186 10.1038/nature03797

[b42] Dong C. . Crystal structure and mechanism of a bacterial fluorinating enzyme. Nature 427, 561–565 (2004). 14765200 10.1038/nature02280

[b43] Wuosmaa A. & Hager L. Methyl chloride transferase: a carbocation route for biosynthesis of halometabolites. Science 249, 160–162 (1990). 2371563 10.1126/science.2371563

[b44] Fetzner S. & Lingens F. Bacterial dehalogenases: biochemistry, genetics, and biotechnological applications. Microbiol. Rev. 58, 641–685 (1994). 7854251 10.1128/mr.58.4.641-685.1994 PMC372986

[b45] Borodina E., McDonald I. R. & Murrell J. C. Chloromethane-dependent expression of the *cmu* gene cluster of *Hyphomicrobium chloromethanicum*. Appl. Environ. Microbiol. 70, 4177–4186 (2004). 15240299 10.1128/AEM.70.7.4177-4186.2004 PMC444766

[b46] McAnulla C. . Chloromethane utilization gene cluster from *Hyphomicrobium chloromethanicum* Strain CM2T and development of functional gene probes to detect halomethane-degrading bacteria. Appl. Environ. Microbiol. 67, 307–316 (2001). 11133460 10.1128/AEM.67.1.307-316.2001 PMC92571

[b47] Alvarez-Cohen L. & Speitel G. E. Kinetics of aerobic cometabolism of chlorinated solvents. Biodegradation 12, 105–126 (2001). 11710590 10.1023/a:1012075322466

[b48] Field J. A. & Sierra-Alvarez R. Biodegradability of chlorinated solvents and related chlorinated aliphatic compounds. Rev. Environ. Sci. Biotechnol. 3, 185–254 (2004).

[b49] McCarty P. L. Breathing with Chlorinated Solvents. Science 276, 1521–1522 (1997). 9190688 10.1126/science.276.5318.1521

[b50] Redon P.-O., Jolivet C., Saby N. P. A., Abdelouas A. & Thiry Y. Occurrence of natural organic chlorine in soils for different land uses. Biogeochemistry 114, 413–419 (2012).

[b51] Breider F. & Albers C. N. Formation mechanisms of trichloromethyl-containing compounds in the terrestrial environment: A critical review. Chemosphere 119, 145–154 (2015). 24974224 10.1016/j.chemosphere.2014.05.080

[b52] IUSS Working Group WRB. World Reference Base for Soil Resources 2014 - International soil classification system for naming soils and creating legends for soil maps. (FAO, 2015).

[b53] Ad-hoc-AG Boden. Bodenkundliche Kartieranleitung. (Federal Institute for Geosciences and Natural Resources, 2005).

[b54] Orsini M. & Romano-Spica V. A microwave-based method for nucleic acid isolation from environmental samples. Lett. Appl. Microbiol. 33, 17–20 (2001). 11442808 10.1046/j.1472-765x.2001.00938.x

[b55] Zhou J., Bruns M. A. & Tiedje J. M. DNA recovery from soils of diverse composition. Appl. Environ. Microbiol. 62, 316–322 (1996). 8593035 10.1128/aem.62.2.316-322.1996 PMC167800

[b56] Meyer F. . The metagenomics RAST server - a public resource for the automatic phylogenetic and functional analysis of metagenomes. BMC Bioinformatics 9, 386, doi: 10.1186/1471-2105-9-386 (2008). 10.1186/1471-2105-9-386 PMC2563014 18803844

[b57] Cox M. P., Peterson D. A. & Biggs P. J. SolexaQA: At-a-glance quality assessment of Illumina second-generation sequencing data. BMC Bioinformatics 11, 485 (2010). 20875133 10.1186/1471-2105-11-485 PMC2956736

[b58] Gomez-Alvarez V., Teal T. K. & Schmidt T. M. Systematic artifacts in metagenomes from complex microbial communities. ISME J. 3, 1314–1317 (2009). 19587772 10.1038/ismej.2009.72

[b59] Buchfink B., Xie C. & Huson D. H. Fast and sensitive protein alignment using DIAMOND. Nat. Methods 12, 59–60 (2015). 25402007 10.1038/nmeth.3176

[b60] Huson D. H., Mitra S., Ruscheweyh H.-J., Weber N. & Schuster S. C. Integrative analysis of environmental sequences using MEGAN4. Genome Res. 21, 1552–1560 (2011). 21690186 10.1101/gr.120618.111 PMC3166839

[b61] Kanehisa M. & Goto S. KEGG: Kyoto Encyclopedia of Genes and Genomes. Nucleic Acids Res. 28, 27–30 (2000). 10592173 10.1093/nar/28.1.27 PMC102409

[b62] Consortium T. U. UniProt: a hub for protein information. Nucleic Acids Res. 43, D204–D212 (2015). 25348405 10.1093/nar/gku989 PMC4384041

[b63] Passardi F. . PeroxiBase: The peroxidase database. Phytochemistry 68, 1605–1611 (2007). 17544465 10.1016/j.phytochem.2007.04.005

[b64] Parks D. H., Tyson G. W., Hugenholtz P. & Beiko R. G. STAMP: Statistical analysis of taxonomic and functional profiles. Bioinformatics 30, 3123–3124 (2014). 25061070 10.1093/bioinformatics/btu494 PMC4609014

[b65] R Core Team. *R: A language and environment for statistical computing*. (R Foundation for Statistical Computing, 2013). at http://www.R-project.org/.

[b66] Myneni S. C. B. Formation of stable chlorinated hydrocarbons in weathering plant material. Science 295, 1039–1041 (2002). 11799203 10.1126/science.1067153

[b67] Gustavsson M. . Organic matter chlorination rates in different boreal soils: the role of soil organic matter content. Environ. Sci. Technol. 46, 1504–1510 (2012). 22191661 10.1021/es203191r

[b68] Hoekstra E. J., Duyzer J. H., de Leer E. W. & Brinkman U. A. T. Chloroform – concentration gradients in soil air and atmospheric air, and emission fluxes from soil. Atmos. Environ. 35, 61–70 (2001).

[b69] Hoekstra E. J., de Leer E. W. B. & Brinkman U. A. T. Natural formation of chloroform and brominated trihalomethanes in Soil. Env. Sci Technol 32, 3724–3729 (1998).

[b70] Breider F. & Hunkeler D. Investigating Chloroperoxidase-catalyzed formation of chloroform from humic substances using stable chlorine isotope analysis. Environ. Sci. Technol. 48, 1592–1600 (2013). 24377317 10.1021/es403879e

[b71] Fierer N. . Cross-biome metagenomic analyses of soil microbial communities and their functional attributes. Proc. Natl. Acad. Sci. 109, 21390–21395 (2012). 23236140 10.1073/pnas.1215210110 PMC3535587

[b72] Mackelprang R. . Metagenomic analysis of a permafrost microbial community reveals a rapid response to thaw. Nature 480, 368–371 (2011). 22056985 10.1038/nature10576

[b73] Fierer N. . Reconstructing the Microbial Diversity and Function of Pre-Agricultural Tallgrass Prairie Soils in the United States. Science 342, 621–624 (2013). 24179225 10.1126/science.1243768

[b74] Taş N. . Impact of fire on active layer and permafrost microbial communities and metagenomes in an upland Alaskan boreal forest. ISME J. 8, 1904–1919 (2014). 24722629 10.1038/ismej.2014.36 PMC4139727

[b75] Suyama A., Yamashita M., Yoshino S. & Furukawa K. Molecular characterization of the pceA reductive dehalogenase of *Desulfitobacterium* sp. strain Y51. J. Bacteriol. 184, 3419–3425 (2002). 12057934 10.1128/JB.184.13.3419-3428.2002 PMC135124

[b76] Bengtson P., Bastviken D. & Öberg G. Possible roles of reactive chlorine II: assessing biotic chlorination as a way for organisms to handle oxygen stress. Environ. Microbiol. 15, 991–1000 (2013). 22712445 10.1111/j.1462-2920.2012.02807.x

[b77] Bugg T. D. H., Ahmad M., Hardiman E. M. & Rahmanpour R. Pathways for degradation of lignin in bacteria and fungi. Nat. Prod. Rep. 28, 1883–1896 (2011). 21918777 10.1039/c1np00042j

[b78] Messerschmidt A. & Wever R. X-ray structure of a vanadium-containing enzyme: chloroperoxidase from the fungus *Curvularia inaequalis*. Proc. Natl. Acad. Sci. 93, 392–396 (1996). 8552646 10.1073/pnas.93.1.392 PMC40244

[b79] Kirner S. . The non-haem chloroperoxidase from *Pseudomonas fluorescens* and its relationship to pyrrolnitrin biosynthesis. Microbiology 142, 2129–2135 (1996). 8760926 10.1099/13500872-142-8-2129

[b80] Bengtson P., Bastviken D., De Boer W. & Öberg G. Possible role of reactive chlorine in microbial antagonism and organic matter chlorination in terrestrial environments. Environ. Microbiol. 11, 1330–1339 (2009). 19453612 10.1111/j.1462-2920.2009.01915.x

[b81] Barloy-Hubler F., Chéron A., Hellégouarch A. & Galibert F. Smc01944, a secreted peroxidase induced by oxidative stresses in *Sinorhizobium meliloti* 1021. Microbiology 150, 657–664 (2004). 14993315 10.1099/mic.0.26764-0

[b82] Jesenská A., Sedlácek I. & Damborský J. Dehalogenation of haloalkanes by *Mycobacterium tuberculosis* H37Rv and other mycobacteria. Appl. Environ. Microbiol. 66, 219–222 (2000). 10618227 10.1128/aem.66.1.219-222.2000 PMC91809

[b83] Burrows K. J., Cornish A., Scott D. & Higgins I. J. Substrate specificities of the soluble and particulate methane mono-oxygenases of *Methylosinus trichosporium* OB3b. J. Gen. Microbiol. 130, 3327–3333 (1984).

[b84] Fox B. G., Borneman J. G., Wackett L. P. & Lipscomb J. D. Haloalkene oxidation by the soluble methane monooxygenase from *Methylosinus trichosporium* OB3b: mechanistic and environmental implications. Biochemistry (Mosc.) 29, 6419–6427 (1990). 10.1021/bi00479a013 2207083

[b85] Cappelletti M., Frascari D., Zannoni D. & Fedi S. Microbial degradation of chloroform. Appl. Microbiol. Biotechnol. 96, 1395–1409 (2012). 23093177 10.1007/s00253-012-4494-1

[b86] Le Mer J. & Roger P. Production, oxidation, emission and consumption of methane by soils: A review. Eur. J. Soil Biol. 37, 25–50 (2001).

[b87] Laturnus F. . Natural formation and degradation of chloroacetic acids and volatile organochlorines in forest soil. Challenges to understanding. Environ. Sci. Pollut. Res. 12, 233–244 (2005). 10.1065/espr2005.06.262 16137159

[b88] Aeppli C., Bastviken D., Andersson P. & Gustafsson Ö. Chlorine isotope effects and composition of naturally produced organochlorines from chloroperoxidases, flavin-dependent halogenases, and in forest soil. Environ. Sci. Technol. 47, 6864–6871 (2013). 23320408 10.1021/es3037669

