# Supplementary material for: A metagenomic-based survey of microbial (de)halogenation potential in a German forest soil
Source: Sci Rep. 2016 Jun 29;6:28958. doi: 10.1038/srep28958 (PMC4926216; doi:10.1038/srep28958)
Supplement: Supplementary Information [file srep28958-s1.pdf]

A metagenomic-based survey of microbial (de)halogenation potential in a German forest soil

Pascal Weigold[1], Mohamed El-Hadidi[2], Alexander Ruecker[1,3], Daniel H. Huson[2], Thomas Scholten[4], Maik Jochmann[5], Andreas Kappler[1], Sebastian Behrens[6,7]

[1] Geomicrobiology, Center for Applied Geosciences, University of Tuebingen, Germany

[2] Algorithms in Bioinformatics, Center for Bioinformatics, University of Tuebingen, Germany

[3] Current affiliation: Baruch Institute of Coastal Ecology and Forest Science, Clemson University, SC, USA

[4] Soil Science and Geomorphology, Geography, University of Tuebingen, Germany

[5] Instrumental Analytical Chemistry, Faculty of Chemistry, University of Duisburg-Essen, Germany

[6] Department of Civil, Environmental, and Geo- Engineering, University of Minnesota, MN, USA

[7] BioTechnology Institute, University of Minnesota, MN, USA

Supplementary Information

FIGURES

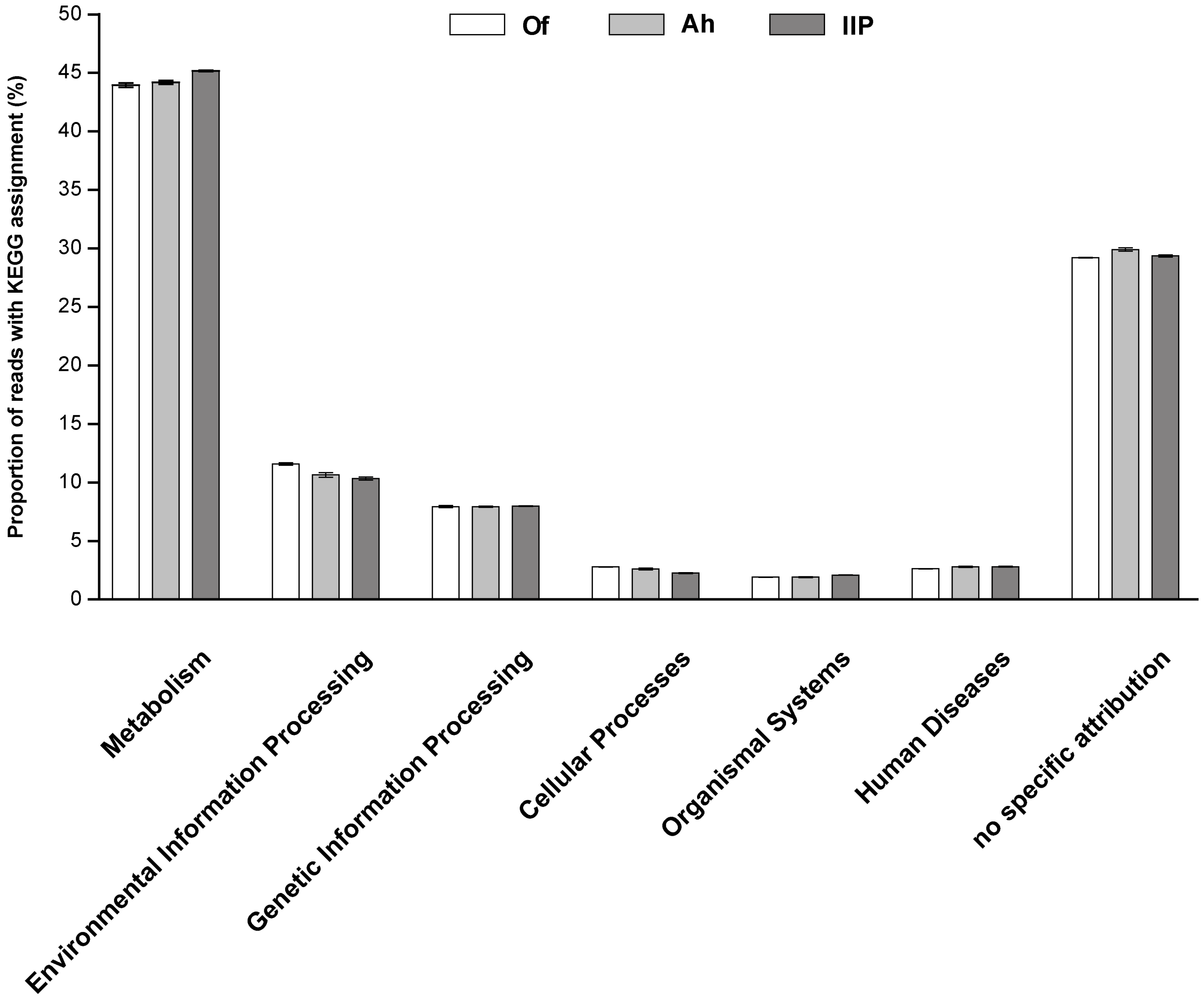

Figure S1 Mean distribution of reads with functional assignment to the KEGG subsystems. Unclassified includes reads related to a KEGG orthology group that is not grouped within one of the subsystems. Error bars indicate standard deviation of the mean for the forward and reverse metagenomic read libraries of duplicate samples for each of the three soil horizons (n=4).

TABLES

Table S1 Sequencing statistics including number of raw reads, post quality processing and annotated reads for the metagenome libraries of the duplicate soil samples. Post QC refers to the number of reads after quality processing by in MG-RAST. Numbers for functional and taxonomic assignment are based on a cutoff of 70% sequence identity on the amino acid level and an e-value of $1x10^{-10}$.

	**Of-1**	**Of-2**	**Ah-1**	**Ah-2**	**IIP-1**	**IIP-2**
Bp count	2.0E+09	2.3E+09	1.7E+09	5.7E+08	1.4E+09	2.2E+09
Raw reads	7,182,766	9,361,450	6,345,700	1,976,590	5,217,938	8,708,660
Post-QC	6,744,172	8,836,157	5,771,696	1,824,150	4,861,826	8,201,432
Annotated reads	1,552,970	1,956,382	1,205,823	365,676	896,863	1,486,485
Assigned taxonomy	1,552,481	1,955,674	1,204,985	365,469	896,514	1,485,746
Assigned KEGG	676,085	868,821	487,240	143,429	365,285	603,453
Annotated reads (%)	23.0	22.1	20.9	20.0	18.4	18.1
Assigned taxonomy (%)	23.0	22.1	20.9	20.0	18.4	18.1
Assigned KEGG (%)	10.0	9.8	8.4	7.9	7.5	7.4

QC: quality control

Table S2 Archaeal and Bacterial 16S rRNA gene copy numbers in the three soil horizons. Values represent the mean and the standard deviation of triplicate measurements for duplicate soil samples for each horizon (n=6) and the standard deviation of the mean.

	**Of**	**Ah**	**IIP**
	**16S rRNA gene copy numbers per g dry soil**		
Archaea	$2.4 \times 10^{8} \pm 1.3 \times 10^{8}$	$7.0 \times 10^{7} \pm 1.9 \times 10^{7}$	$5.5 \times 10^{7} \pm 1.4 \times 10^{7}$
Bacteria	$1.6 \times 10^{11} \pm 1.6 \times 10^{10}$	$5.0 \times 10^{10} \pm 3.8 \times 10^{10}$	$5.6 \times 10^{10} \pm 2.8 \times 10^{10}$

Table S3 Copy numbers of bacterial *nosZ*- and *nifH*-genes, the haloalkane dehalogenase gene of *Mycobacterium smegmatis* strain MC2155 (*dhaA*) and the flavin-dependent halogenase gene (*prnA*) of *Pseudomonas fluorescens* in the three soil horizons. Values represent the mean and the standard deviation of triplicate measurements for duplicate soil samples for each horizon (n=6) and the standard deviation of the mean.

	**Of**	**Ah**	**IIP**
	**gene copy numbers ng^{-1} extracted DNA**		
nosZ	$3.6 \times 10^{2} \pm 1.7 \times 10^{2}$	$5.4 \times 10^{2} \pm 1.6\ 10^{2}$	$5.5 \times 10^{2} \pm 8.8 \times 10^{1}$
nifH	$2.0 \times 10^{6} \pm 2.8 \times 10^{5}$	$1.1 \times 10^{6} \pm 2.2 \times 10^{5}$	$1.6 \times 10^{6} \pm 1.2 \times 10^{5}$
dhaA	$3.7 \times 10^{1} \pm 4.8 \times 10^{0}$	$3.3 \times 10^{1} \pm 1.7 \times 10^{1}$	$1.0 \times 10^{2} \pm 6.9 \times 10^{1}$
prnA	$6.3 \times 10^{0} \pm 3.9 \times 10^{0}$	$1.9 \times 10^{0} \pm 5.2 \times 10^{-1}$	$6.3 \times 10^{0} \pm 3.9 \times 10^{2}$

SUPPLEMENTARY METHODS

Quantitative PCR of bacterial and archaeal 16S rRNA genes

Quantitative PCR (qPCR) for archaeal and bacterial 16S rRNA genes, typical nitrous oxide reductase genes (*nosZ*), bacterial nitrogenase genes (*nifH*), the haloalkane dehalogenase gene of *Mycobacterium smegmatis* (*dhaA*) and the flavin-dependent halogenase gene (*prnA*) of *Pseudomonas fluorescens* was performed on an iQ5 real-time PCR detection system (iQ5 optical system software, version 2.0, Bio-Rad). Primer sequences and thermal profiles are displayed in Tables S4 and S5, respectively.

For archaeal and bacterial 16S rRNA genes 20 µL reaction volumes contained 1× SsoFast™ Eva Green® Supermix (Bio-Rad Laboratories GmbH, Munich, Germany), 2 µL of a 1:100 diluted DNA extract and 75 nM of primer 341F and 225 nM of primer 797R for bacterial 16S rRNA genes or 250 nM of each archaeal 16S rRNA gene primers. Reactions for all functional genes were performed in 10 µL reaction volumes containing 1x SsoAdvanced™ SYBR® Green Supermix (Bio-Rad Laboratories GmbH, Munich, Germany), 1 µL of diluted DNA extract (1:10 or 1:100) and 250 nM of each primer. For *prnA* each reaction volume contained 0.1 µL DMSO (≥99.5%) and for dhaA each reaction volume contained 0.1 µL DMSO (≥99.5%) and 0.5 µM Betaine. Each qPCR was followed by a melt curve analysis.

Table S4 Target genes and primers used for quantitative PCR.

Gene	Primer	Sequence (5'-3')	Amplicon size (bp)	Reference
archaeal	109F	ACKGCTCAGTAACACGT	806	[1]
16S rRNA	915R	GTGCTCCCCCGCCAATTCCT		[2]
bacterial	341F	CCTACGGGAGGCAGCAG	456	[3]
16S rRNA	797 R	GGACTACCAGGGTATCTAATCCTGTT		[4]
nosZ	nosZ2F	CGCRACGGCAASAAGGTSMSSGT	267	[5]
	nosZ2R	CAKRTGCAKSGCRTGGCAGAA		
nifH	nifHF	AAAGGYGGWATCGGYAARTCCACCAC	458	[6]
	nifHR	TTGTTSGCSGCRTACATSGCCATCAT		
dhaA	Ms_HAD_F	CGCATGTGATCTGATCGGGA	152	this study
	Ms_HAD_R	CCCAGTCGTGTAGTACGAGC		
prnA	prnA_A_F	GGAATGGATGCCCCAAGTGA	115	this study
	prnA_A_R	CACGTTGCCGAACAAATGGT		

Table S5 Thermal profiles and reference genes of quantitative PCR

Gene	Thermal profile	Reference strain	Cloning vector
archaeal<br>16S rRNA	1: 98°C - 10 min<br>2: 40 cycles<br>98°C - 5 s<br>52°C - 12 s<br>72°C - 15 s	*Halobacterium salina*	pCR™ 4®
bacterial<br>16S rRNA	1: 98°C - 2 min<br>2: 40 cycles<br>98°C - 5 s<br>60°C - 12 s	*Thiomonas* sp.	pCR™ 2.1®
nosZ	1: 98°C - 2 min<br>2: 40 cycles<br>98°C - 15 s<br>60°C - 25 s	*Ensifer meliloti* strain 1021	pCR™ 4®
nifH	1: 98°C - 2 min<br>2: 45 cycles<br>98°C - 30 s<br>55°C - 30 s<br>72°C - 30 s	*Acidithiobacillus ferrooxidans*	pCR™ 4®
dhaA	1: 98°C - 2 min<br>2: 40 cycles<br>98°C - 15 s<br>55°C - 20 s	*Mycobacterium smegmatis*	pEX-A2
prnA	1: 98°C - 2 min<br>2: 40 cycles<br>98°C - 15 s<br>57°C - 20 s	*Pseudomonas fluorescens*	pEX-A2

REFERENCES

1. Großkopf, R., Janssen, P. H. & Liesack, W. Diversity and structure of the methanogenic community in anoxic rice paddy soil microcosms as examined by cultivation and direct 16S rRNA gene sequence retrieval. *Appl. Environ. Microbiol.* **64,** 960–969 (1998).
2. Amann, R. & Stahl, D. A. in *Nucleic acid techniques in bacterial systematics* 205–248 (John Wiley & Son Ltd, 1991).

3. Muyzer, G., Teske, A., Wirsen, C. & Jannasch, H. Phylogenetic relationships of *Thiomicrospira* species and their identification in deep-sea hydrothermal vent samples by denaturing gradient gel electrophoresis of 16S rDNA fragments. *Arch. Microbiol.* **164,** 165–172 (1995).
4. Nadkarni, M. A., Martin, F. E., Jacques, N. A. & Hunter, N. Determination of bacterial load by real-time PCR using a broad-range (universal) probe and primers set. *Microbiology* **148,** 257–266 (2002).
5. Henry, S., Bru, D., Stres, B., Hallet, S. & Philippot, L. Quantitative detection of the nosZ gene, encoding nitrous oxide reductase, and comparison of the abundances of 16S rRNA, *narG*, *nirK*, and *nosZ* genes in soils. *Appl. Environ. Microbiol.* **72,** 5181–5189 (2006).
6. Rösch, C., Mergel, A. & Bothe, H. Biodiversity of denitrifying and dinitrogen-fixing bacteria in an acid forest soil. *Appl. Environ. Microbiol.* **68,** 3818–3829 (2002).
